# Initial Experience with Lecanemab and Lessons Learned in 71 Patients in a Regional Medical Center

**DOI:** 10.14283/jpad.2024.159

**Published:** 2024-09-03

**Authors:** L. B. E. Shields, H. Hust, S. D. Cooley, G. E. Cooper, R. N. Hart, B. C. Dennis, S. W. Freeman, J. F. Cain, W. Y. Shang, K. M. Wasz, A. T. Orr, C. B. Shields, S. S. Barve, Kenneth G. Pugh

**Affiliations:** 1 Norton Healthcare, Norton Neuroscience Institute, Louisville, KY 40202 USA; 2 Department of Neurological Surgery, University of Louisville School of Medicine, Louisville, KY 40202 USA; 3 Department of Medicine, University of Louisville School of Medicine, Louisville, KY 40202 USA; 4 Norton Healthcare, Norton Neuroscience Institute Memory Center, Norton Medical Plaza III- Brownsboro, Suite 300, 4915 Norton Healthcare Blvd., Louisville, KY 40241 USA

**Keywords:** Alzheimer’s disease, mild cognitive impairment, lecanemab, amyloid-related imaging abnormalities

## Abstract

**Background and Objectives:**

On July 6, 2023 the U.S. Food and Drug Administration approved the anti-amyloid monoclonal antibody lecanemab (Leqembi®) for treatment of patients with mild cognitive impairment or mild dementia due to Alzheimer’s disease (AD). Our early experience and lessons learned with lecanemab in a regional community medical center are described.

**Design, Setting, and Participants:**

This retrospective observational study highlights the first 71 patients treated with lecanemab at our multidisciplinary Norton Neuroscience Institute Memory Center. All patients had positive cerebrospinal fluid biomarkers for AD and underwent at least 1 lecanemab infusion. Two patients had additional amyloid PET scans which were positive.

**Results:**

The mean age was 72 years (49–90 years), and 44 (62%) patients were female. Most were Caucasian (68 [96%]), and 54 [76%] were referred to our Memory Center by their primary care provider. Comorbidities were common, including hypertension (34 [48%]), hypercholesterolemia (51 [72%]), diabetes mellitus (17 [24%]), and cardiovascular disease excluding hypertension (22 [31%]). The mean body mass index was 27.0 (range: 17.8–45.0). A total of 36 (51%) patients were heterozygous for the ApoE4 genotype, and 9 (13%) were homozygous. A total of 61 [86%] patients had been treated with donepezil; 40 (56%) patients had received memantine. Of the 50 patients who completed 1 or more safety monitoring brain MRIs following infusion, 12 (24%) had amyloid-related imaging abnormalities (ARIA) detected: solitary ARIA-H (hemorrhage) in 5, solitary ARIA-E (edema) in 3, and both ARIA-H and ARIA-E in 4. Of the 12 patients with ARIA, 9 were asymptomatic, 4 were homozygous for the ApoE4 genotype, and 6 were heterozygous for the ApoE4 genotype. Of the 9 who were homozygous for the ApoE4 genotype in this study, 4 (44%) had evidence of ARIA. Of the 36 who were heterozygous for the ApoE4 genotype, 6 (17%) were diagnosed with ARIA. Twenty-six (37%) patients experienced infusion reactions after their first lecanemab infusion: headaches (12 patients) and shaking/chills/rigors (11 patients) were most common. Twenty-three (88%) of these 26 patients reported the side effects either at the infusion center or within the first 24 hours post-infusion. One patient died shortly after the first lecanemab infusion of a myocardial infarction. It is uncertain whether or not this death was related to lecanemab treatment.

**Conclusion:**

Through our early experience with lecanemab, we have recognized several areas of improvement which have clarified and enhanced the lecanemab infusion experience.

## Introduction

Lecanemab (Leqembi®) is a recombinant humanized immunoglobulin gamma 1 (IgG1) monoclonal antibody that binds with high affinity to amyloid beta (Aβ) soluble protofibrils (1–7). The U.S. Food and Drug Administration granted lecanemab accelerated approval on January 6, 2023 and traditional approval on July 6, 2023 to treat patients with mild cognitive impairment (MCI) or mild dementia due to Alzheimer’s disease (AD) who have confirmed brain amyloid pathology (1, 8, 9). This approval was based on promising results from phase II and III trials (4, 5, 7). In Van Dyck and colleagues’ 18-month, multicenter, doubleblind, phase III Clarity AD trial with 1795 patients (898 treated with lecanemab and 897 with placebo), lecanemab reduced markers of amyloid in early AD and resulted in moderately less decline on measures of cognition and function than placebo at 18 months (7). Interpretation of the phase III Clarity AD trial demonstrated that 68% of treated individuals experienced complete clearance of Aβ by amyloid PET imaging at 18 months (8). Additionally, the lecanemab-treated group experienced a statistically significant 27% reduction in decline on the primary outcome measure, the Clinical Dementia Rating (CDR®) Scale sum of boxes, after 18 months of treatment. In Qiao and colleagues’ systematic review and meta-analysis of 4 randomized controlled trials (3108 AD patients: 1695 treated with lecanemab, 1413 control) receiving lecanemab for AD, significant positive statistical efficacy with respect to cognition, function, and behavior in patients with early AD were observed, though actual clinical significance has yet to be established (10).

While lecanemab has demonstrated favorable results in phase II and III trials in treating MCI or mild dementia due to AD, several dose-dependent adverse events have been reported, consisting primarily of amyloid-related imaging abnormalities (ARIA) and infusion-related reactions (4, 5, 7). ARIA-E (edema) refers to MR signal alterations representing vasogenic edema or sulcal effusion of proteinaceous material, while ARIA-H (hemorrhage) denotes MR signal alterations attributable to microhemorrhages, macrohemorrhages, and hemosiderosis (superficial siderosis) (6, 11). Careful monitoring for ARIA-E and ARIA-H on brain MRIs and being alert to associated symptoms (headaches, dizziness, and increasing confusion) while being treated with lecanemab is imperative (1). Infusion-related adverse reactions often involve fever, chills, nausea, vomiting, headaches, rash, abdominal discomfort, and elevated blood pressure (1, 6, 12). The latter usually occur during the lecanemab infusion or up to several hours after the infusion, resolving within 24 hours (1). They are most common before the third biweekly lecanemab infusion (1).

We describe the first 71 patients who were treated with lecanemab for MCI and mild dementia due to AD in a regional, metropolitan community medical center. We have acknowledged and followed the American Association of Neurology guidelines and the appropriate use recommendations for the prescribing of anti-amyloid therapies in patients with AD (1, 13). The adverse events associated with lecanemab (ARIA-H/ARIA-E and infusion-related side effects) are discussed. Several lessons learned from our initial experience with lecanemab are also presented.

## Methods

### Study population

Under an Institutional Review Board-approved protocol and according to the Declaration of Helsinki, we performed a retrospective review of the first 71 patients treated with lecanemab at the Norton Neuroscience Institute Memory Center (NNI-MC) over 6 months (August 25, 2023–March 1, 2024). All patients underwent at least 1 infusion of lecanemab during this time period. Inclusion criteria included patients with (1) MCI or mild dementia due to AD and (2) amyloid positivity of CSF AD biomarkers or by amyloid PET scan. If a patient remained independent in activities of daily living (ADLs), then MCI was diagnosed. If the history confirmed impairment in ADLs (mostly instrumental ADLs), then the patient was characterized as mild AD dementia. The clinical interview and informant corroboration were used to distinguish these conditions. The CSF tests were performed using the Roche Elecsys® assay ptau 181/Aβ42 ratios above the cutpoint from the manufacturer. We utilized the Mayo Clinic lab. The amyloid PET scans were done using AMYVID tracer and were visually read as positive or negative. The baseline pre-lecanemab Memory MRI protocol consisted of sagittal T1 and Magnetization-Prepared Rapid Gradient-Echo (MP-RAGE) images, along with axial T2, FLAIR, and susceptibility weighted imaging (SWI). MRI scans used to image for potential ARIA were a sagittal T1 with axial Diffusion-weighted Imaging (DWI) and T2, FLAIR, and SWI.

The exclusion criteria consisted of (1) severe vascular dementia (severe cerebrovascular changes on MRI or multiple lacunar strokes or cortical infarcts), (2) more than 4 microhemorrhages (10 mm or less at the greatest diameter) or a single macrohemorrhage (greater than 10 mm at the greatest diameter), (3) history of active cancer, (4) severe psychiatric or depressive disorders, (5) currently pregnant, (6) poorly controlled immunologic disorders, (7) unstable medical conditions, (8) stroke, TIA, bleeding disorders, or seizures in the previous 12 months, (9) cerebral amyloid angiopathy-related inflammation/amyloid beta-related angiitis, (10) currently receiving aducanumab or have had severe or recurrent ARIA with aducanumab, and (11) warfarin, vitamin K antagonists, direct oral anticoagulants, heparin, acute thrombolytics, and clotting disorders. Antiplatelet therapy was allowed. Patients with other neurodegenerative disorders such as dementia with Lewy bodies were excluded based on clinical evaluation and CSF biomarkers that did not suggest AD.

**Figure 1:**
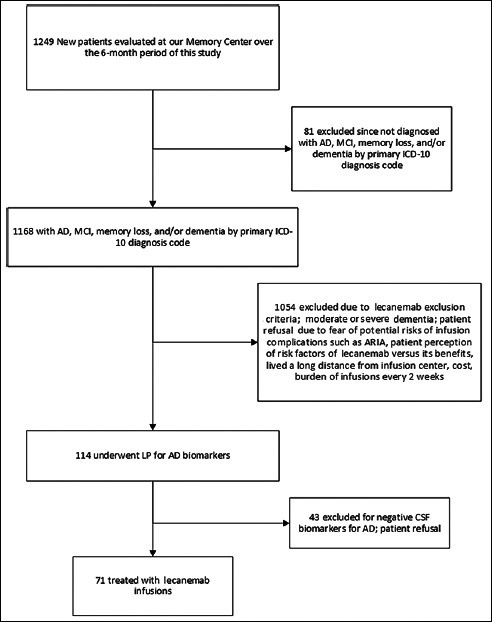
Algorithm of the screening process for lecanemab at our Memory Center over the 6-month period of this study AD: Alzheimer’s disease; MCI: mild cognitive impairment; LP: lumbar puncture; CSF: cerebrospinal fluid

**Figure 2:**
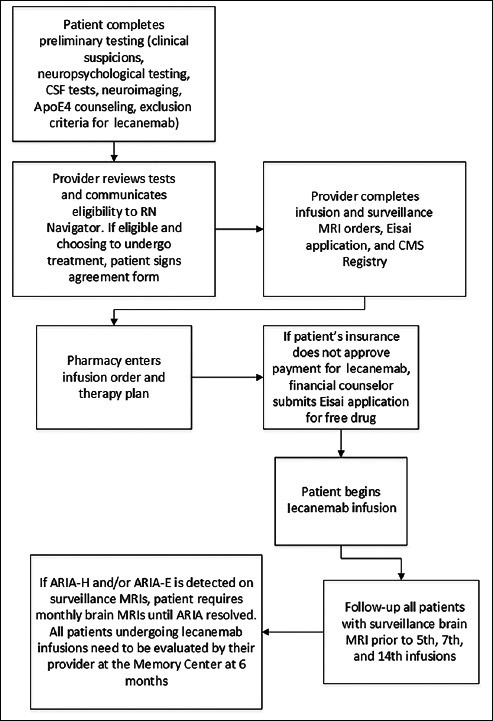
Timeline of the patient journey from initial evaluation to start of lecanemab therapy and follow-up

Figure 1 depicts an algorithm of the screening process for lecanemab at our Memory Center. Patients may have been excluded from receiving lecanemab by either the provider or the patient. The patient may have been excluded based on the lecanemab exclusion criteria or having MoCa/MMSE scores that were too low. At the start of our study, no patients declined lecanemab following the LP for Alzheimer’s biomarkers. As the study progressed, approximately 15% of patients turned down lecanemab for a host of reasons, including cost, the burden of infusions every 2 weeks, living too far from the infusion center, fear of the potential risks of infusion complications such as ARIA, and patient perception of the risk factors of lecanemab versus its benefits. Figure 2 summarizes the timeline of the patient journey from initial evaluation to start of lecanemab therapy and follow-up.

The NNI-MC features a multidisciplinary team comprised of neurologists, geriatricians, advanced practice providers, neuropsychologists, neurosurgeons, pharmacists, social workers, and hospital administrators. All patients complete a clinical evaluation including cognitive testing (Mini-Mental State Examination [MMSE] or Montreal Cognitive Assessment [MoCA]), brain MRI, and review of history to identify exclusion criteria prior to AD biomarker testing. More extensive neuropsychological testing was performed when clinically indicated and/or when specifically requested by insurance carriers as part of the authorization process. All patients were required to undergo ApoE genotyping so that we would be able to discuss the potential greater risks of ApoE4 if the patients were homozygous for ApoE4. Patients are treated with intravenous (IV) lecanemab 10 mg/kg per infusion with an infusion every 2 weeks. Baseline brain MRIs are performed prior to the initial lecanemab administration, and then surveillance MRIs are obtained before the 5th, 7th, and 14th infusions (7). If ARIA-H and/or ARIA-E was detected on surveillance MRIs, the patient required monthly MRIs until ARIA resolved. Patients have a 6-month follow-up appointment with their memory care provider. We currently have two infusion centers where lecanemab is administered. The nurse navigator, who was hired by our Institution, visits each patient on the first infusion date during which education about lecanemab and the infusion process is reviewed. Possible lecanemab side effects, what to do if an adverse event occurs, our on-call system for off-hour events, and other information about the treatment process are discussed. The nurse navigator contacts patients by phone one day following the initial lecanemab infusion to assess their condition.

Several metrics were collected including the patient’s age, gender, race, Body Mass Index (BMI), highest level of education and employment, medical specialty of referring physician, comorbidities, payer of lecanemab, MMSE/MoCA scores, post-lumbar puncture spinal headaches, ApoE genotype, medications for MCI/AD (donepezil, memantine, galantamine, rivastigmine), ARIA-H and/or ARIA-E on surveillance brain MRIs, and infusion-related side effects. The severity of ARIA-H and ARIA-E were also determined (14, 15).

### ARIA Protocol and Management

Our recommendations for ARIA management with lecanemab were based on the phase III Clarity AD trial (7) and the lecanemab appropriate use recommendations (1). Table 1 highlights the protocol and management of ARIA at our Memory Center.

**Table 1:** ARIA Protocol and Management at our Memory Center

**ARIA Protocol**	**ARIA Management**
- Neuroradiologist notifies provider of suspected ARIA on surveillance brain MRI - Neuroradiologist classifies degree of ARIA based on appropriate use criteria severity measures -Provider reviews and concurs with neuroradiologist - Provider contacts patient to explain MRI results, discusses any changes in plan of care - Provider documents MRI results, ARIA, updated plan of care, and discussion with patient/caregiver - Provider contacts nurse navigator of ARIA and any updates to plan of care - Nurse navigator contacts pharmacy, infusion center, MRI scheduler, and finance to update plan of care - Nurse navigator awaits results of next MRI and plan of care from provider - Nurse navigator updates all departments of next steps	- Our recommendations for ARIA management with lecanemab are based on phase III Clarity AD trial and lecanemab appropriate use recommendations * - Review of symptoms and possible in person examination of symptoms related to ARIA - To detect ARIA early and mange it safely, radiologists and treating clinicians must recognize and interpret the pattern of MRI changes as well as recognize and manage the associated symptoms - Treatment with lecanemab can continue for individuals with radiographically mild asymptomatic ARIA recognized only on MRI with careful monitoring for symptoms and monthly non-contrast MRI scans until ARIA-E has resolved and/or ARIA-H has stabilized - For those with moderate or severe radiographic ARIA or those with symptoms, lecanemab infusions should be suspended; patients should receive careful clinical monitoring, management of symptoms, and monthly non-contrast MRI until ARIA-E resolves and/or ARIA-H stabilizes; once ARIA symptoms and radiographic ARIA-E changes resolve, restarting lecanemab can be considered based on a discussion of risks and benefits with the patient and family - Recurrent ARIA can occur; decisions regarding ARIA management and when to discontinue treatment require consideration of the severity of the radiographic changes, presence and severity of any symptoms, patient’s APOE genotype, co-morbidities, and concurrent medications

AUR: appropriate use recommendations; * van Dyck CH, Swanson CJ, Aisen P, et al. Lecanemab in early Alzheimer’s disease. N Engl J Med. 2023;388:9–21; doi: 10.1056/NEJMoa2212948; * Cummings J, Apostolova L, Rabinovici GD, et al. Lecanemab: appropriate use recommendations. J Prev Alzheimers Dis. 2023;10:362–377; doi: 10.14283/jpad.2023.30

### Ethical approval and informed consent

The WCG Institutional Review Board (IRB) determined that this retrospective study was exempt under 45 CFR 46.104(d)(4). The IRB number is 2024-0001, and the IRB approval was dated January 3, 2024. According to federal regulations, the IRB of record determined that this study was exempt Category 4 with a complete waiver of consent and authorization.

## Results

### Clinical characteristics

Of the 71 patients who were treated with lecanemab, 36 (50.7%) patients were considered mild dementia and 35 (49.3%) were considered MCI. The mean age at the first lecanemab infusion was 72 years (49–90 years), and 44 (62%) patients were female (Table 2). A total of 68 (96%) patients were Caucasian, and 54 (76%) of patients were referred to our Memory Center by their primary care provider. Only 4 (6%) patients did not complete high school; 20 (28%) had a post-college degree. Comorbidities were common, including hypertension (34 [48%]), hypercholesterolemia (51 [72%]), diabetes mellitus (17 [24%]), and cardiovascular disease excluding hypertension (22 [31%]). The mean body mass index was 27.0 (range: 17.8–45.0). Thirty-six (51%) patients were heterozygous for the ApoE4 genotype, and 9 (13%) were homozygous for ApoE4. Of the 71 patients treated with lecanemab, 55 (77%) were assigned only a MMSE score, 28 (39%) received only a MoCA score, and 12 (17%) had both tests performed. The mean MMSE score was 25.0; the mean MoCA score was 20.2. Four patients had initial MoCA scores less than 18, and 1 patient had an initial MMSE score less than 20. Two patients with MoCA scores less than 18 (11 and 7) had corresponding MMSE scores of 20 and 19, respectively.

**Table 2:** Clinical Characteristics and Adverse Events of Patients Treated with Lecanemab at our Memory Center

**Features**	**Category**	**Number of Patients (n=71)**
Diagnosis	Mild dementia MCI	36 (50.7%) 35 (49.3%)
Age at 1st lecanemab infusion (mean)		72 years (Range: 49–90 years)
Gender	Male	27 (38%)
	Female	44 (62%)
Race	Caucasian	68 (96%)
	African-American	2 (3%)
	Asian	1 (1%)
Body Mass Index		Mean: 27.0 (Range: 17.8–45.0)
	< 19.9	3 (4%)
	20.0–24.9	18 (25%)
	25.0–29.9	40 (56%)
	30.0–34.9	7 (10%)
	35.0–39.9	1 (1%)
	> 40.0	2 (3%)
Highest level of education	Did not complete high school	4 (6%)
	High school graduate	15 (21%)
	Some college	7 (10%)
	Technical school after high school	4 (6%)
	Associate’s degree	2 (3%)
	College graduate	18 (25%)
	Post-college degree *	20 (28%)
	Unknown	1 (1%)
Medical specialty of referring physician	Primary Care Provider	54 (76%)
	Self-referral	11 (15%)
	Neurologist	6 (8%)
Hypertension	Yes	34 (48%)
	No	37 (52%)
Hypercholesterolemia	Yes	51 (72%)
	No	20 (28%)
Diabetes mellitus	Yes	17 (24%)
	No	54 (76%)
Cardiovascular disease (other than hypertension)	Yes	22 (31%)
	No	49 (69%)
Payer of lecanemab	Medicare	47 (66%)
	Patient Assistance Program	18 (25%)
	Commercial insurance	6 (8%)
ApoE genotype	E4 heterozygous	36 (51%)
	E4 homozygous	9 (13%)
	Other	26 (37%)
Pathology assessment of CSF biomarkers	p-Tau/Abeta42 ratio	Mean: 0.063
	Abeta42	Mean: 650.6 pg/mL
	Total-Tau	Mean: 377.4 pg/mL
	Phospho-Tau	Mean: 46.5 pg/mL
MMSE/MoCA Scores (mean) (55 had MMSE, 28 had MoCA, 12 had both)	MMSE score	25.0 (Range: 19–30)
	MoCA score	20.2 (Range: 7–26)
Medications for mild cognitive impairment/AD	Donepezil	61 (86%)
	Memantine	40 (56%)
	Galantanime	3 (4%)
	Rivastigmine	3 (4%)
	None	4 (6%)
Post-lumbar puncture headaches (64 performed at our Institution)	Yes	3 (5%) (2 required blood patch)
	No	61 (95%)
Findings on baseline brain MRI	Microhemorrhages	7 (9.9%)
	Superficial siderosis	1 (1.4%)
	Periventricular/subcortical hyperintensities	64 (90.1%)
	Mild	46 (71.9%)
	Moderate	15 (23.4%)
	Severe **	3 (4.7%)
ARIA-H/ARIA-E detected on surveillance brain MRI (50 had at least 1 surveillance brain MRI)	Yes	12 (24%)
	Solitary ARIA-H	5 (42%)
	Solitary ARIA-E	3 (25%)
	Both ARIA-H and ARIA-E	4 (33%)
	No	38 (76%)
Infusion-related adverse effects following 1st lecanemab infusion and when they started	Yes	26 (37%)
	At Infusion Center (0–3 hours)	10 (38%)
	3–24 hours post-infusion	13 (50%)
	24–48 hours post-infusion	2 (8%)
	48–72 hours post-infusion	1 (4%)
	No	45 (63%)

MCI: Mild cognitive impairment; AD: Alzheimer’s disease; * Post-college degree: Master’s, PhD, MD, JD; ** 3 patients had white matter changes on the baseline MRI that were initially read as “advanced”, “prominent”, and “moderate to severe”, respectively, by radiologists outside of our institution which we initially documented as “severe”. Upon further review of the brain MRI scans by the treating neurologists and neuroradiologist at our Institution prior to the initiation of lecanemab, it was determined that 2 of these patients had mild white matter disease and 1 patient had moderate white matter disease. None was found to have Fazekas grade 3 white matter disease.

A total of 61 (86%) patients had been treated with donepezil; 40 (56%) patients had received memantine. Medicare was the payer in 47 (66%) of cases. A total of 13 patients missed lecanemab doses, 5 of whom missed doses due to the detection of ARIA on surveillance MRI. The other 8 patients missed doses for a variety of reasons, including other concurrent medical treatments (a breast lumpectomy in 1 patient and an infusion for osteoporosis in another patient), a change of insurance, a vacation planned on the date of the infusion, having missed a mandatory surveillance MRI prior to the infusion, and not feeling well.

### Findings on baseline brain MRI

On baseline brain MRI, 7 (9.9%) patients had microhemorrhages (3 patients had 1, 3 patients had 2, 1 patient had 3), and 1 (1.4%) patient had 2 foci of superficial siderosis each measuring less than 1 cm in diameter. A total of 64 (90.1%) patients had evidence of subcortical hyperintensities on baseline MRI that were either mild (46 [71.9%]), moderate (15 [23.4%]), or severe (3 [4.7%]).

### Pathology assessment of CSF biomarkers and amyloid PET scan results

All patients underwent CSF testing for AD biomarkers. The mean p-Tau/Abeta42 ratio was 0.063 (Reference value: ≤ 0.028), the mean Abeta42 was 650.6 pg/mL (Reference value: > 834 pg/mL), the mean Total-Tau was 377.4 pg/mL (Reference value: ≤ 238 pg/mL), and Phospho-Tau was 46.5 pg/mL (Reference value: ≤ 21.6 pg/mL). Two patients underwent amyloid PET scans which were positive.

### ARIA-H and ARIA-E detected on surveillance brain MRI

**Figure 3:**
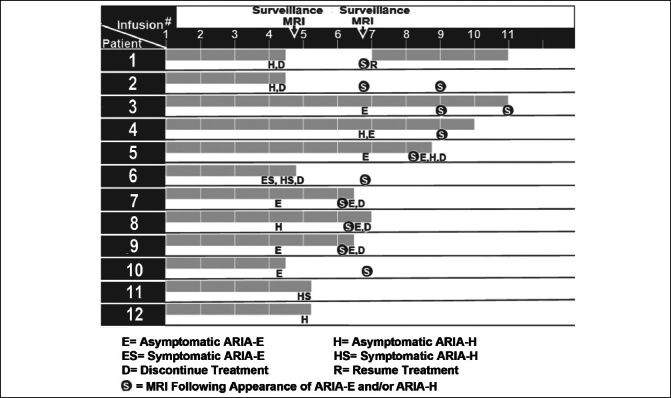
Initial detection of ARIA-H and ARIA-E on brain MRI scans in relation to the number of lecanemab infusions

Of the 50 patients who completed 1 or more safety monitoring brain MRIs following infusion, 12 (24%) had amyloid-related imaging abnormalities (ARIA) detected: solitary ARIA-H (hemorrhage) in 5, solitary ARIA-E in 3, and both ARIA-H and ARIA-E in 4 (Tables 2 and 3, Figure 3). Of the 12 patients who had evidence of ARIA, 9 were asymptomatic and 2 were treated with aspirin 81 mg per day. Two symptomatic patients reported headaches, while the fourth complained of fatigue and disorientation. Seven patients had either temporary or permanent suspension of lecanemab infusions due to either persistent ARIA on multiple brain MRIs, a large number of ARIA foci on a single MRI, and/or associated symptoms. Of the 12 patients with ARIA, 4 were homozygous for the ApoE4 genotype and 6 were heterozygous for the ApoE4 genotype. Of the 9 patients who were homozygous for the ApoE4 genotype in this study, 4 (44%) had evidence of ARIA. Consistent with prior evidence, this finding reflects the substantially increased risk of ARIA with a homozygous ApoE4 genotype. Of the 36 patients who were heterozygous for the ApoE4 genotype, 6 (17%) were diagnosed with ARIA. Two patients underwent unscheduled safety MRIs, neither of whom had ARIA detected. These unscheduled safety MRIs were performed following the complaint of vertigo in 1 patient and headaches in another patient.

**Table 3:** ARIA-H and ARIA-E in the Patients Treated with Lecanemab at our Memory Center

**Patient #**	**Age/Gender**	**Co-morbidities**	**APOE4 Genotype**	**Brain MRI Prior to Lecanemab Infusion**	**Brain MRIs During Lecanemab Infusions**	**Lecanemab Treatment Status**	**Severity of ARIA/Symptomatic**
1	67/M	HTN, HC, DM	e4/e4	2 foci cerebral microhemorrhages	Before 5th infusion: 3 new foci ARIA-H (microhemorrhages)	Lecanemab suspended; 1st MRI following ARIA negative for new ARIA- H and ARIA-E; lecanemab resumed	Moderate ARIA-H/No
2	80/M	HTN, HC, DM, CVD *	e3/e4	2 foci superficial siderosis each less than 1 cm in diameter	Before 5th infusion: 9 new foci ARIA-H (microhe-morrhages), unchanged appearance of superficial siderosis (less than 1 cm in diameter)	Lecanemab suspended; 1st MRI following ARIA: 1 new focus ARIA-H (microhemorrhage), unchanged appearance of superficial siderosis (less than 1 cm in diameter); 2nd MRI following ARIA: 1 new focus ARIA-H (microhemorrhage); lecanemab not resumed	Moderate ARIA-H on 1st surveillance MRI; severe ARIA-H on 1st and 2nd MRI following the onset of ARIA/No
3	72/M	HC, DM, CVD **; Aspirin 81 mg	e3/e4	No evidence of microhemorrhages or vasogenic edema	Before 7th infusion: 1 focus ARIA-E (8.0 mm in diameter)	Lecanemab continued; 1st MRI following ARIA negative for new ARIA-H and ARIA-E, previous focus of ARIA-E resolved	Mild ARIA-E/No
4	77/F	HTN	e3/e3	No evidence of microhemorrhages or vasogenic edema	Before 7th infusion: 4 new foci ARIA-H (microhemorrhages) and 1 new focus ARIA-E (4.0 cm in diameter)	Lecanemab continued; 1st MRI following ARIA negative for new ARIA-H, decreased size of ARIA-E (3.7 cm in diameter), and 1 new infarct (7.0 mm in diameter) in the left cerebellar hemisphere	Mild ARIA-H and ARIA-E/No
5	74/M	HC	e3/e3	No evidence of microhemorrhages or vasogenic edema	Before 7th infusion: 1 focus ARIA-E (4.2 cm in diameter) and 1 focus of ARIA-H (microhemorrhage)	Lecanemab continued; 1st MRI following ARIA: 1 new focus ARIA-E (less than 1 cm in diameter) and increased size of previous focus of ARIA-E (5.8 cm in diameter); lecanemab suspended	Mild ARIA-E on 2nd surveillance MRI, moderate ARIA-E on 1st MRI following the onset of ARIA/No
6	71/F	None; Aspirin 81 mg	e4/e4	No evidence of microhemorrhages or vasogenic edema	Before 5th infusion: 6 foci ARIA-H (microhemorrhages) and 1 new focus ARIA-E (6.0 cm in diameter)	Lecanemab suspended; 1st MRI following ARIA: 5 new ARIA-H (microhemorrhages) and decrease in size of previous ARIA-E (3.0 cm)	Moderate ARIA-H and moderate ARIA-E on 1st surveillance MRI, severe ARIA-H and mild ARIA-E on 1st MRI following the onset of ARIA/Yes (headaches)
7	71/F	HC	e3/e4	No evidence of microhemorrhages or vasogenic edema	Before 5th infusion: 1 focus ARIA-E (less than 1 cm in diameter)	Lecanemab continued until after 5th infusion when suspended due to HA and history of ARIA-E	Mild ARIA-E/Yes (headaches)
8	75/F	HTN, HC	e4/e4	No evidence of microhemorrhages or vasogenic edema	Before 5th infusion: 1 focus ARIA-H (microhemorrhage)	Lecanemab continued; 1st MRI following ARIA negative for new ARIA-H and ARIA-E	Mild ARIA-H/No
9	74/F	HTN, HC	e3/e4	No evidence of microhemorrhages or vasogenic edema	Before 5th infusion: 1 focus ARIA-E (1.8 cm in diameter)	Lecanemab continued; 1st MRI following ARIA: increased size of ARIA-E (2.2 cm in diameter) and 1 new focus ARIA-E (3.0 cm); lecanemab suspended	Mild ARIA-E on 1st surveillance MRI, Moderate ARIA-E on 1st MRI following the onset of ARIA/No
10	73/F	HC	e4/e4	No evidence of microhemorrhages or vasogenic edema	Before 5th infusion: 1 focus ARIA-E (3.0 cm in diameter) and 4 new ARIA-H (microhemorrhages)	Lecanemab suspended; 1st MRI following ARIA: increased size of ARIA-E (3.4 cm) and 4 new foci of ARIA-H (microhemorrhages)	Mild ARIA-E and mild ARIA-H on 1st surveillance MRI; fatigue/disorientation for several days after 4th infusion; mild ARIA-E and moderate ARIA-H on 1st MRI following the onset of ARIA
11	71/F	HC	e2/e4	No evidence of microhemorrhages or vasogenic edema	Before 5th infusion, 2 new foci ARIA-H (microhemorrhages)	Lecanemab continued	Mild ARIA-H/No
12	74/F	None	e2/e4	No evidence of microhemorrhages or vasogenic edema	Before 5th infusion, 2 new foci ARIA-H (microhemorrhages)	Lecanemab continued	Mild ARIA-H/No

* History of deep venous thrombosis, congestive heart failure, superficial venous thrombosis; ** History of cardiovascular small vessel disease; ARIA-H: amyloid-related imaging abnormalities-hemorrhage; ARIA-E: amyloid-related imaging abnormalities-edema; HTN: hypertension; HC: hypercholesterolemia; DM: diabetes mellitus; e4: APOE4; e3: APOE3

Before the 7th lecanemab infusion, Patient 4 in Table 3 had evidence of 4 new foci of ARIA-H and 1 new focus of ARIA-E (4.0 cm in diameter) on 1st surveillance MRI. Lecanemab was not discontinued. The 1st MRI following ARIA was negative for new ARIA-H, decreased size of ARIA-E (3.7 cm in diameter), and 1 new infarct measuring 7.0 mm in diameter in the left cerebellar hemisphere which had not been identified on a prior MRI. The lecanemab was suspended after ARIA was detected in both Cases 6 and 10 in Table 3 who were homozygous for ApoE4. Both patients required monthly MRIs until the ARIA resolves. These patients will resume lecanemab infusions if the ARIA-H and ARIA-E resolve.

### Infusion-related side effects

A total of 26 (37%) patients experienced infusion-related side effects after their first lecanemab infusion: headaches (12 patients) and shaking/chills/rigors (11 patients) were most common (Tables 2,4). Twenty-three (88%) of these 26 patients reported the side effects either at the infusion center [0–3 hours] (10 patients) or between 3–24 hours post-infusion (13 patients). Two patients were evaluated in the Emergency Department (ED) for infusion-related adverse events (both with headaches) after the first infusion.

**Table 4:** Infusion-Related Side Effects at our Memory Center

**Infusion #**	**Number of Patients who Underwent Infusion and had Infusion-Related Side Effects**	**Infusion-Related Side Effects**	Number of Specific Infusion-Related Side Effects *
1	1st infusion: 71/71 patients IRSE after 1st infusion: 26/71 (37%) patients	Headaches Shaking/chills/rigors Body aches Nausea Pain of lower extremities Vomiting Hypertension Fever Mouth blisters Diarrhea Subjective weakness Unsteady gait Gastric irritation Dizziness	12 (46%) 11 (42%) 4 (15%) 3 (12%) 2 (11%) 2 (8%) 2 (8%) 2 (8%) 1 (4%) 1 (4%) 1 (4%) 1 (4%) 1 (4%) 1 (4%)
2	2nd infusion: 67/71 (94%) patients IRSE after 2nd infusion: 5/67 (7%) patients	Headaches Shaking Nausea Vomiting Dyspnea Pain of lower extremities	2 (40%) 1 (20%) 1 (20%) 1 (20%) 1 (20%) 1 (20%)
3	3rd infusion: 58/71 (82%) patients IRSE after 3rd infusion: 3/58 (5%) patients	Headaches Shaking Flu-like symptoms Dizziness “Getting lost” at night/confusion (no h/o ARIA on brain MRI)	1 (33%) 1 (33%) 1 (33%) 1 (33%) 1 (33%)
4	4th infusion: 50/71 (70%) patients IRSE after 4th infusion: 2/50 (4%) patients	Headaches Chills Fever Dizziness	1 (50%) 1 (50%) 1 (50%) 1 (50%)
5	5th infusion: 41/71 (58%) patients IRSE after 5th infusion: 2/41 (5%) patients	Headaches Balance problems	2 (100%) 1 (50%)
6	6th infusion: 31/71 (44%) patients IRSE after 6th infusion: 2/31 (6%) patients	Leg pain Dizziness	1 (50%) 1 (50%)
7	7th infusion: 27/71 (38%) patients IRSE after 7th infusion: 1/27 (4%) patients	Body aches	1 (100%)
8	8th infusion: 22/71 (31%) patients IRSE after 8th infusion: 0/22 (0%) patients	None	
9	9th infusion: 20/71 (28%) patients IRSE after 9th infusion: 0/20 (0%) patients	None	
10	10th infusion: 17/71 (24%) patients IRSE after 10th infusion: 0/17 (0%) patients	None	
11	11th infusion: 12/71 (17%) patients IRSE after 11th infusion: 0/12 (0%) patients	None	
12	12th infusion: 7/71 (10%) patients IRSE after 12th infusion: 0/7 (0%) patients	None	
13	13th infusion: 2/71 (3%) patients IRSE after 13th infusion: 0/2 (0%) patients	None	
14	14th infusion: 1/71 (1%) patients IRSE after 14th infusion: 0/1 (0%) patients	None	

* A single patient may have experienced more than one infusion-related side effect; IRSE: infusion-related side effects

Of the 67 patients who received a second lecanemab infusion, 5 (7%) complained of infusion-related side effects (2 with headaches). One patient had a 1-night hospital stay due to dyspnea 3 days following the second infusion. One patient was evaluated in the ED after the third infusion for dizziness. A cranial CT showed no evidence of acute hemorrhage, and her symptoms resolved after being treated with IV fluids. Another patient was seen at the ED for leg pain after the sixth infusion. An abdominal/pelvic CT was normal, and pain medications were prescribed with resolution of symptoms. Infusion-related adverse events were reported in 37% of patients after the 1st infusion, 7% after the 2nd infusion, 4–6% between the 3rd and 7th infusions, and none between the 8th and 14th infusions.

Two patients experienced serious behavioral reactions after taking diphenhydramine to treat the infusion-related adverse events early in our lecanemab treatment program. One patient was given diphenhydramine for rigors and subsequently exhibited confusion, agitation, belligerence, and a hostile behavior. Another patient was also given diphenhydramine for fever and chills, after which he sustained a fall and repeatedly banged his head against a door. These episodes prompted us to revise the pretreatment medication protocol for infusion-related adverse events.

### Death following lecanemab infusion

There was one patient death during the lecanemab infusion time frame described in this paper. A 75-year-old woman with a history of hypertension, hypercholesterolemia, and diabetes mellitus underwent her first lecanemab infusion, after which she remained in the infusion center for 3 hours for observation with no complications. She was homozygous for the ApoE4 genotype, had a MMSE score of 24, and was taking daily donezepil 10 mg and memantine 10 mg. The patient did not have a history of coronary artery disease or cardiac arrhythmia. She had been advised to discontinue the monoclonal antibody denosumab (Prolia) 60 mg/mL which she had last taken 4 ½ months prior to the infusion. A 3.0T brain MRI prior to lecanemab revealed no evidence of ARIA, however, a remote small vessel infarction in the left cerebellum was noted. On being driven home from the infusion, she complained of leg pain, nausea, and vomiting followed by a syncopal episode and cardiac arrest. Cardiopulmonary resuscitation was performed, and EMS arrived at the scene within 10 minutes. She was initially in ventricular fibrillation, after which two defibrillation procedures were administered. Asystole ensued, and then 4 rounds of epinephrine and 100 mg lidocaine were given.

The patient was transferred to the closest ED. Upon arrival, the pupils were fixed and dilated; the Glasgow Coma Scale score was 3. A 12-lead ECG revealed atrial fibrillations with a slow ventricular response, right bundle branch block, left anterior fascicular block, and marked ST abnormality. These findings indicated a possible lateral infarct or inferior subendocardial injury. No intracranial hemorrhage was detected on brain CT shortly before death. A chest x-ray showed mild pulmonary edema. Her death was confirmed 12 hours after the lecanemab infusion. The diagnoses were cardiorespiratory arrest and anoxic brain injury. An autopsy was not performed. After a thorough analysis of the circumstances surrounding the patient’s death, our Memory Center determined that her death was most likely cardiac-related, however, the association with lecanemab could not be excluded. This patient’s death was reported to both the manufacturer and the U.S. Food and Drug Administration (FDA).

## Discussion

### Lessons Learned

Several lessons have been learned from our initial experience with lecanemab in our patient population which may be divided into the following categories: (1) organizational, (2) clinical, and (3) payment and insurance coverage (Table 5).

**Table 5:** Lessons Learned During Our Initial Use of Lecanemab

**Organizational**	**Clinical**	**Payment and Insurance Coverage**
Create a multidisciplinary planning team to prepare and create protocols	Prophylactic treatment consisting of acetaminophen, loratadine, and famotidine prior to lecanemab infusion to reduce the number of side effects	Streamline the Medicare/commercial insurance payment of lecanemab
Weekly conferences for the memory center team to review patients who have been treated with lecanemab and those who will be undergoing lecanemab infusion	Long distances to our Memory Center for some patients	
Value of a lecanemab coordinator to oversee details of the study and being available for phone calls from patients/families	Caregivers must drive patients to all lecanemab infusion appointments	
Educate other physicians about the potential adverse events of lecanemab	Caregivers must remain with patient for entire first lecanemab infusion	
Standardize radiology reporting before and after lecanemab to monitor safety	Ascertain whether the patient is taking monoclonal antibodies	
How to avoid and manage post-lumbar puncture headaches following diagnostic lumbar punctures for biomarkers	Strongly recommend that all patients have a 3T MRI prior to their first lecanemab infusion	
Identification and management of lecanemab patients in the Emergency Department		
All charts of lecanemab patients flagged in our electronic medical record system		
Familiarity with entering patients into a lecanemab database		
Medical bracelets for lecanemab patients		
Need to expand sites for lecanemab infusion		
Value of a lecanemab caregivers/patient support group		

### Lessons Learned: Organizational

Our Memory Center created a multidisciplinary planning team to prepare and create lecanemab protocols. This planning team was established 7–8 months before administering our 1st lecanemab infusion. Our Memory Center team attends weekly conferences to review patients who have been treated with lecanemab and those who will be undergoing lecanemab infusion. The lecanemab nurse navigator plays an invaluable role in overseeing details of the treatment protocol and being available for phone calls from patients/families. Additionally, the nurse navigator is important for monitoring the patients who may miss lecanemab doses. All patients are required to sign a consent agreement prior to initiating lecanemab, stating that they will be compliant with the anticipated 18 months of lecanemab treatment. Although patients consent to being compliant with lecanemab, certain circumstances may arise that necessitate their missing a lecanemab dose. Our Memory Center has educated other physicians about the potential adverse events of lecanemab, such as general neurologists who may be on call and are asked to evaluate patients’ MRI scans post-lecanemab, as well as emergency medicine physicians, radiologists, and intensive care specialists. We have also standardized radiology reporting before and after starting lecanemab to monitor safety by identifying amyloid-related imaging abnormalities (14).

Our Memory Center has addressed the occurrence of post-lumbar puncture headaches following diagnostic lumbar punctures for biomarkers (16), including the use of a 22G spinal needle instead of the customary 20G spinal needle. Special attention was also made to use polypropylene low-binding tubes and collect the appropriate volume of CSF for biomarkers of AD to ensure accurate interpretation of biomarkers for AD (17, 18).

We developed a protocol to identify and manage lecanemab patients in the ED. Conventional ED protocol involves ordering a head CT for a symptom such as a headache looking for intracranial hemorrhage. However, lecanemab patients require a brain MRI to assess for potential ARIA. Since we have implemented this protocol, the number of CTs inappropriately ordered has decreased. Additionally, we have flagged all charts of lecanemab patients in our electronic medical record system and have become familiar with entering patient data into the lecanemab CMS database. We have also discussed a medical bracelet, stating that the patient is on lecanemab which may be especially valuable if a patient is evaluated at an outlying ED. A mild bottleneck that we have encountered is the infusion center capacity. Due to the high demand for lecanemab, we needed to expand from one to two infusion sites. We have not outsourced the lecanemab infusions outside of our hospital system yet. We have not faced any bottlenecks with respect to access to LP procedures or imaging.

Our Memory Center developed a lecanemab patient/caregiver support group that is an important component of the lecanemab experience. This interactive support group offers patients a platform to share their experiences and an opportunity to support each other. Numerous topics have been discussed during the lecanemab support group meetings including: managing a new diagnosis (MCI or mild dementia due to AD), the mechanisms of lecanemab, vitamins/foods to help with memory, exercise and brain health, forming a walking group, travel and billing questions, and developing a prevention clinic for family members.

### Lessons Learned: Clinical

When our Memory Center started treating patients with lecanemab, we observed a large number of infusion-related adverse events. Therefore, diphenhydramine prophylaxis prior to the lecanemab infusion was initiated in some patients to curtail the side effects; this medication was subsequently discontinued due to adverse behavioral reactions. Starting on December 21, 2023, every patient was pretreated at the infusion center before every infusion with an oral cocktail of acetaminophen 650 mg, loratadine 10 mg, and famotidine 20 mg. Our infusion center had used this same cocktail for pretreating patients undergoing other monoclonal antibody infusions. Therefore, we adopted this pretreatment protocol for lecanemab. The pretreatment cocktail has greatly decreased the number of infusion-related side effects. Of the 40 patients whose 1st lecanemab infusion occurred before December 21, 2023, 18 (45%) had infusion-related adverse events. Of the 31 patients whose 1st infusion occurred after the implementation of the pretreatment cocktail, 8 (26%) experienced infusion-related adverse events. Prior to pretreating all patients receiving lecanemab, only patients who experienced an infusion-related adverse event were pretreated. Additionally, patients who were undergoing lecanemab infusions prior to the current pretreatment medication triad and not experiencing infusion-related adverse events were not pretreated with the cocktail.

Three patients had white matter changes on the baseline MRI that were initially read as “advanced”, “prominent”, and “moderate to severe”, respectively, by radiologists outside of our Institution which we initially documented as “severe”. Upon further review of the brain MRI scans by the treating neurologists and neuroradiologist at our Institution prior to the initiation of lecanemab, it was determined that 2 of these patients had mild white matter disease and 1 patient had moderate white matter disease. None was found to have Fazekas grade 3 white matter disease. Therefore, these patients were eligible for lecanemab treatment. We have realized the importance of having the treating neurologists and neuroradiologist at our Institution review all baseline MRIs that were initially read by radiologists outside of our Institution prior to starting lecanemab.

The patient with the initial MoCA score of 7 had an MMSE score of 19 on the same day. This patient subsequently underwent repeat MMSE testing 3 months later prior to starting lecanemab which demonstrated a score of 22. Even though her initial MoCA score was low and may have represented an outlier, her treating neurologist at our Memory Center opined that she presented functionally with mild dementia, justifying her treatment with lecanemab. We learned it is vital to not evaluate the MoCA score as an independent factor in making a decision about lecanemab treatment and should be evaluated in conjunction with other tests performed concomitantly. It is also important to repeat MoCA/MMSE testing prior to initiating lecanemab especially if there is a large discrepancy between the MoCA and MMSE scores on the same day.

While we recognize the increased risk of ARIA in patients who are homozygous for the ApoE4 genotype, we are continuing to treat them with lecanemab. These patients are followed very closely throughout treatment by monthly MRI scans following the appearance of ARIA. The 2 foci of superficial siderosis on baseline brain MRI were not initially detected by the radiologist. Upon further review of all cases by a neuroradiologist for the purpose of this study, the superficial siderosis was recognized. Patients receiving lecanemab travel from across our state up to 2 hours to receive the infusion as it cannot be administered at an off-site facility. A caregiver must drive the patient to and from all infusions as well as being present throughout the entire first lecanemab infusion in case the patient experiences an infusion-related adverse reaction. We also ascertained whether patients are taking other monoclonal antibodies. It is suggested that patients need to discontinue monoclonal antibodies, including denosumab (Prolia), prior to starting lecanemab.

Due to the importance of detecting ARIA-H and ARIA-E during lecanemab treatment and to avoid variability in post-treatment reporting, all patients should preferably undergo a 3.0T MRI scan prior to initiating this drug. If patients present to our Memory Center with a 1.5T MRI, a 3.0T MRI is required. As all of the surveillance brain MRIs are performed on a 3.0T MRI, it is important to obtain a similar strength scan with the same vendor prior to the start of lecanemab to be able to identify new ARIA post-lecanemab.

Even if a patient is eligible for lecanemab, we have observed that he/she may choose not to proceed with infusions for several reasons: (1) long distance from the infusion site; (2) large time commitment; (3) homozygous for the ApoE4 genotype with a known higher risk of ARIA-H and ARIA-E; (4) concern for adverse events especially ARIA against the perception of a relatively modest benefit; and (5) finances (some patients have excessive out-of-pocket costs even with insurance).

### Lessons Learned: Payment and Insurance Coverage

The payment of lecanemab posed one the biggest hurdles and warranted streamlining of this process. When the Centers for Medicare and Medicaid Services (CMS) issues a new National Coverage Determination (NCD) for Medicare coverage of a newly approved drug, most commercial insurers have adhered to Medicare’s decision, although specific policies pertaining to copays, coinsurance, prior authorization, and step therapy may vary (19). However, this has not occurred with lecanemab as more than half of the national private insurance companies with a publicly available policy are denying coverage. Medicare does not require an authorization, thus, patients with Medicare are scheduled for infusions without difficulty in our experience. However, patients with Medicare Advantage plans have faced difficulties to obtain lecanemab approval, with a large number of appeal letters and peer-to-peer reviews required (19). Medicare Advantage plans are required to cover all of the services that original Medicare covers except hospice care. It should be noted that funding differs in different countries and healthcare systems.

We did not encounter any difficulties with commercial payers regarding payment for CSF testing for AD confirmation. ApoE testing was covered by insurance. We did not order any amyloid PET scans in any of the 71 patients in our study. The 1 patient who had an amyloid PET scan had it performed outside of our facility. For those patients who received drug from the manufacturer, the ancillary costs (infusions, CSF testing, imaging) were covered by the patients’ insurance company.

Some insurance carriers requested additional cognitive or functional measures, including the CDR®, which was administered as part of the Clarity AD trial but which is not commonly used in clinical practice. In other cases, they requested specific test scores, such as from the Weschler Memory Scale – Fourth Edition, Logical Memory I & II subtests. To our knowledge, this may be the first instance in which approval for disease modifying therapy has been contingent on specific neuropsychological test scores. These requirements and strict adherence to the clinical trial procedures seem to reflect the novelty and the potentially transformative nature of this new class of drug in the fight against AD. This will also limit real-world availability at some institutions due to availability. Other commercial insurance companies balk at the high frequency of MRIs, especially if (1) the patient has evidence of ARIA on brain MRI and requires monthly MRIs; (2) a patient has 2 MRIs within one month (before the 5th and 7th infusions); or (3) an additional 3.0T MRI is required after the patient has already had a 1.5T MRI before lecanemab initiation. Some plans may deny coverage for patients having additional neurologic or mental health conditions that may contribute to dementia (19). It has been reported that all commercial plans also require documentation of clinical improvement (specific score ranges on the CDR® and on at least one of the MMSE, MoCA, or Saint Louis University Mental Status [SLUMS]) for continuation of lecanemab past the initial 6 months (19). Eight commercial payers do not cover lecanemab stating that the “clinical benefit has not been established” (19).

We provide the requested clinical documentation when peer-to-peer consultation is required. If we receive 2 insurance denials, the patient will be evaluated for free drug through Eisai’s Patient Assistance Program (PAP). Another common reason why patients may be on free drug is the high out-of-pocket costs despite the drug being approved by their insurance company or payer. All patients have been able to receive the lecanemab, however, their initial treatment has often been delayed as we pursued the appeal process. To date, the manufacturer Eisai has granted 18 patients with free drug.

### Data Presented

Our study concurs with numerous findings in the phase III Clarity AD trial (7), including comparable percentages of our infusion-related reactions (37% of patients after the 1st infusion), most (88%) occurring within 24 hours of the infusion, and the majority following the first infusion and decreasing in frequency with subsequent infusions (37% of patients following the 1st infusion which decreased to 5% of patients by the 3rd infusion). Headaches were also the most common adverse side effect in our study. The majority of cases of ARIA in our study were also asymptomatic (75%), and most (75%) ARIA occurred during the first 3 months of the initial infusion. A high percentage (33%) of patients who demonstrated ARIA were homozygous for the ApoE4 genotype.

Although patients in our study may have been initially assigned a MoCA score in the lower end of range, patients subsequently underwent neuropsychological testing that made them eligible for lecanemab infusion. The increase in score may be attributed to either starting donezepil or memantine or discontinuing a medication that may have suppressed cognitive function. Lecanemab trials permitted the simultaneous administration of symptomatic anti-dementia therapies (choninesterase inhibitors and memantine) (1). In the phase III Clarity AD trial, 52% of patients who were treated with lecanemab were also treated with medications for symptoms of AD (7). Even though memantine is not approved for early AD, there is widespread use of memantine in our community in an attempt to improve cognitive function. Memantine was also prescribed to patients who have failed other treatment methods or who have experienced toxic effects from donezepil such as gastrointestinal symptoms.

### Fatalities Associated with Lecanemab

Few deaths have been reported that were associated with lecanemab (7, 8, 20–22). In the phase III Clarity AD trial, 13 deaths occurred which were evenly distributed between treated and placebo groups (0.7% of participants in the lecanemab group and 0.8% of those in the placebo group) (7). In the open-label extension phase of their study, 3 brain hemorrhage-related deaths were attributed to lecanameb (combined with anticoagulants in 2 patients and combined with IV tissue plasminogen activator for an acute cerebrovascular accident (CVA) in 1 patient) (7, 8, 21). In this latter case, the 65-year-old patient was homozygous for the ApoE4 genotype and had received 3 previous infusions of lecanemab, with the last infusion 4 days prior to the CVA. A brain MRI performed 81 days before the CVA demonstrated mild small-vessel disease without microhemorrhages or edema. A repeat MRI 3 days after the CVA revealed an acute right thalamocapsular ischemic infarction and innumerable multifocal cortical and subcortical hemorrhages with surrounding edema. An autopsy showed extensive multifocal intraparenchymal hemorrhages, cerebral amyloid angiopathy, and diffuse histiocytic vasculitis with necrotizing vasculopathy involving amyloid deposition within the blood vessel walls. The patient had no contraindication to thrombolysis. Solopova and colleagues reported another of these 3 cases, specifically, a 79-year-old woman who experienced a seizure (speech arrest and generalized convulsions) after the third lecanemab infusion (22). She was homozygous for the ApoE4 genotype and had complained of headaches following each of her 3 lecanemab infusions. A brain MRI revealed multifocal swelling and a marked increase in the number of cerebral microhemorrhages. An autopsy showed severe cerebral amyloid angiopathy with perivascular lymphocytic infiltrates, reactive macrophages, fibrinoid degeneration of vessel walls, as well as deposits of β-amyloid in the meningeal vessels and penetrating arterioles with numerous microaneurysms. The cause of death was attributed to severe cerebral amyloid-related inflammation. Following these 3 reported lecanemab-related fatalities, it was hypothesized that lecanemab weakened the blood vessels in the brain as it attacked the amyloid plaques lining them (20). We are unable to determine whether any of these neurovascular findings may have contributed to the patient’s death in our paper since an autopsy was not performed.

### Strengths and Limitations of the Current Study

Our study featuring 71 patients with MCI and mild dementia due to AD treated with lecanemab is the largest reported to date in a regional community medical center. In contrast to the phase II and III lecanemab trials with strict eligibility criteria, our study describes the implementation of lecanemab in a real-world metropolitan community setting. The first 6 months of lecanemab administration have presented a rewarding learning experience for our Memory Center. By recognizing the initial financial and radiological obstacles as well as high number of infusion-related side effects, we developed strategies to minimize the infusion-related adverse events through the preinfusion cocktail, streamline the payment process, and standardize the radiology process. The protocols and management of lecanemab as well as lessons learned from our experience may be applicable to other regional medical facilities who are planning to initiate a lecanemab program to treat patients with AD. Limitations of our work include the modest patient numbers, short follow-up, low diversity of the patient population which potentially limits the generalizability of our study findings, and no follow-up for neurocognitive function. The rate of ARIA-E and ARIA-H may be underestimated in the current study as some patients had not received surveillance MRIs prior to the 5th and 7th lecanemab infusion if they were treated with their first dose of lecanemab shortly before the end of the 6-month duration of our study. Additionally, we realize the importance of the ALZ-NET network developed by the Alzheimer’s Association to track safety and efficacy of amyloid-lowering treatments for AD, however, we did not use this network during the current study due to the delay in obtaining IRB approval to share information. We have just received IRB approval and will initiate implementing it on a daily basis in our Memory Center.

In conclusion, lecanemab was well-tolerated by most patients with MCI and mild dementia due to AD. By identifying numerous areas of improvement and implementing solutions, the lecanemab infusion experience for patients was enhanced. Providers treating patients with amyloid-lowering antibody medications should select patients most likely to benefit with a clearly positive risk-benefit profile.
